# Overexpression of 1-Aminocyclopropane-1-Carboxylic Acid Deaminase (*acdS*) Gene in *Petunia hybrida* Improves Tolerance to Abiotic Stresses

**DOI:** 10.3389/fpls.2021.737490

**Published:** 2021-11-02

**Authors:** Aung Htay Naing, Hui Yeong Jeong, Sung Keun Jung, Chang Kil Kim

**Affiliations:** ^1^Department of Horticultural Science, Kyungpook National University, Daegu, South Korea; ^2^Forest Medicinal Resources Research Center, NIFoS, Yeongju, South Korea; ^3^School of Food Science and Biotechnology, Kyungpook National University, Daegu, South Korea

**Keywords:** abiotic stress, ACC deaminase, bedding plants, ethylene production, gene expression, plant growth

## Abstract

Abiotic stress induces the ethylene precursor 1-aminocyclopropane-1-carboxylic acid (ACC) in plants, which consequently enhances ethylene production and inhibits plant growth. The bacterial ACC deaminase enzyme encoded by the *acdS* gene reduces stress-induced ethylene production and improves plant growth in response to stress. In this study, overexpression of *acdS* in *Petunia hybrida* (‘Mirage Rose’) significantly reduced expression of the ethylene biosynthesis gene ACC oxidase 1 (*ACO1*) and ethylene production relative to those in wild type (WT) under various abiotic stresses (cold, drought, and salt). The higher reduction of stress-induced ethylene in the transgenic plants, which was due to the overexpression of *acdS*, led to a greater tolerance to the stresses compared to that in the WT plants. The greater stress tolerances were proven based on better plant growth and physiological performance, which were linked to stress tolerance. Moreover, expression analysis of the genes involved in stress tolerance also supported the increased tolerance of transgenics relative to that with the WT. These results suggest the possibility that *acdS* is overexpressed in ornamental plants, particularly in bedding plants normally growing outside the environment, to overcome the deleterious effect of ethylene on plant growth under different abiotic stresses. The development of stress-tolerant plants will be helpful to advance the floricultural industry.

## Introduction

Ethylene is regarded as an important regulator of many plant developmental and physiological processes (from seed germination to senescence; Pierik et al., 2006; Masood et al., 2012; Nazar et al., 2014). In addition, it is also known as a stress-induced phytohormone because plants induce ethylene inside their tissues and organs in response to biotic and abiotic stresses, including cold, drought, and salt stressors (Arshad and Frankenberger, 2002). Moreover, it is used as a signaling molecule in stress tolerance mechanisms to protect plants from the deleterious effects of stressors (Lin et al., 2009; Van de Poel et al., 2015; Wen, 2015). However, higher concentrations of ethylene inside plant tissues cause senescence by promoting aging and reductions in chlorophyll, as well as the retardation of plant growth by inhibiting root elongation (Grichko and Glick, 2001; Glick, 2014), which can negatively affect crop yield. As global climate change has recently become more severe, the frequent occurrence and persistence of abiotic stresses are expected in the near future, which will result in more adverse effects on plant growth *via* the generation of reactive oxygen species (ROS) and the overproduction of ethylene (Bournonville and Diaz-Ricci, 2011; You and Chan, 2015; Assaha et al., 2017). The adverse effects of ethylene production on plant growth for several plant species under drought stress have been demonstrated using biotechnological approaches (Graves and Gladon, 1985; Balota et al., 2004; Young et al., 2004; Shi et al., 2012, 2015; Habben et al., 2014; Zhao et al., 2014). Compared to wild-type (WT) characteristics, mutants with ethylene biosynthetic genes knocked-out or knocked-down or transgenic plants overexpressing an ethylene inhibitory gene were found to have lower ethylene production and maintain plant growth under drought stress (Young et al., 2004; Habben et al., 2014; Shi et al., 2015). Freezing tolerance associated with the reduction of ethylene production is due to the suppression of cold-regulated genes *via* cold-enhanced ethylene production (Shi et al., 2012; Zhao et al., 2014).

Ethylene is derived from methionine, which is converted into S-adenosyl-L-methionine (SAM) by SAM synthetase. SAM is then converted to 1-aminocyclopropane-1-carboxylic acid (ACC) by ACC synthetase. Then, ACC is finally converted to ethylene by ACC oxidase (Yang and Hoffman, 1984; Barnawal et al., 2014). Plant growth-promoting bacteria (PGPB) produce the ACC deaminase enzyme, which can break down ACC into ammonia and α-ketobutyrate in all higher plants (Honma and Shimomura, 1978; Glick, 2014). Therefore, plants inoculated with PGPB, such as *Pseudomonas putida*, *Sinorhizobium meliloti*, and *Medicago lupulina*, can are protected from the more severe conditions caused by abiotic stresses *via* the reduction of ethylene overproduction and alleviation of the ethylene-mediated adverse effect on plant growth *via* the action of ACC deaminase (Grichko and Glick, 2001; Mayak et al., 2004; Glick, 2005, 2014; Safronova et al., 2006; Belimov et al., 2009; Kong et al., 2015; Tavares et al., 2018). ACC deaminase is encoded by the *acdS* gene, and many researchers have isolated these genes from various PGPB and introduced them into different plant species. Compared to that with WT plants, the overexpression of *acdS* remarkably improves plant growth *via* a reduction in ethylene overproduction, alleviating the negative effect of ethylene on plants (Reed et al., 1995; Sergeeva et al., 2006; Farwell et al., 2007; Zhang et al., 2008; Jung et al., 2018).

*Petunia* has become increasingly popular in the landscape industry because of its diversity in flower shapes and colors. However, since these plants are sensitive to ethylene, their flower longevity and plant growth are associated with an increase in ethylene production (Woltering and van Doorn, 1988; Tang et al., 1993; Tang and Woodson, 1996; Huang et al., 2007). Moreover, as a type of bedding plant, they might be extremely susceptible to ethylene induced by abiotic stresses, which are not readily controllable in nature. As described, introduction of the *acdS* gene, which encodes ACC deaminase, into plants significantly reduces ethylene production and improves plant growth in response to various biotic and abiotic stresses (Reed et al., 1995; Sergeeva et al., 2006; Farwell et al., 2007; Zhang et al., 2008; Jung et al., 2018). However, to our knowledge, the *acdS* gene of *Petunia* has not been well investigated to date despite its importance as a bedding plant in the landscape industry.

Therefore, we introduced the *acdS* gene isolated from *Pseudomonas veronii*-KJ into *Petunia hybrida*, ‘Mirage Rose’, using *Agrobacterium*-mediated transformation methods. The transgenic *Petunia* overexpressing the *acdS* gene was exposed to various abiotic stresses, such as cold, salinity, and drought stressors, and tolerance to abiotic stresses was investigated by evaluating plant growth, ethylene content, transcriptional activation of ethylene biosynthetic genes, and other genes related to abiotic stresses.

## Materials and Methods

### Plant Material

Seeds of *P. hybrida* cv. ‘Mirage Rose’ were surface-sterilized in 70% ethanol for 30s, followed by immersion in a 0.5% sodium hypochlorite solution for 5min. After this, seeds were washed at least three times in sterilized water and blotted dry on filter paper. Following this, the seeds were sown on a hormone-free Murashige and Skoog (MS) basal medium containing 3.0% of sucrose and 0.7% of plant agar, and they were placed in a culture condition set up with a temperature (25°C±2°C) and 16h photoperiod (intensity, 35μmolm^−2^ s ^−1^) for 4weeks. The 4-week-old *in vitro* seedlings were sub-cultured on the same hormone-free MS medium for another 6weeks, and healthy plants with uniform sizes were chosen as explant sources for *Agrobacterium*-mediated genetic transformation.

### Plasmid Construction

*Agrobacterium tumefaciens* strain GV3101, which harbors the plasmid PCB 302-3, was used in this study. The plasmid was constructed using the *acdS* gene isolated from *P. veronii-KJ* (Jung et al., 2018) in which the *acdS* gene was placed under the control of the cauliflower mosaic virus 35S (CaMV 35S) promoter, and the *bar* gene conferring phosphinothricin (PPT) resistance was used as a selection marker.

### *Agrobacterium*-Mediated Genetic Transformation

Genetic transformation of *Petunia* leaf explants was performed as described by Ai et al. (2017), with some modifications. Briefly, approximately 500 leaf explants excised from 6-week-old *Petunia* plants were pre-cultured in a shoot regeneration medium. The pre-cultured explants were infected with an *Agrobacterium* suspension, which was adjusted to an OD_600_ of 0.6, and incubated at room temperature for 30min. Following this, the explants were blotted on sterile filter paper and cultured in co-cultivation medium in the dark. After 2days of culture, the co-cultivated explants were transferred to a selection medium containing 0.5mg/L PPT, and shoots regenerated from the selection medium were further transferred to PPT (1.0mg/L) containing hormone-free MS medium. Plantlets with roots were removed and washed thoroughly with sterile distilled water before being transferred to plastic pots containing peat-based soil for acclimatization and growth in a greenhouse. The acclimatization process and greenhouse conditions were the same as those described in the work of Ai et al. (2017).

### DNA Extraction and Detection of *acdS* Gene in Transgenic Lines Using PCR

Total genomic DNA was extracted from the leaves of putative transgenic lines using the HiYield^™^ Genomic DNA Mini Kit (Real Biotech Corporation, Taipei, Taiwan). The plasmid PCB 302-3*acdS* was used as a positive control. PCR was performed using the primers for *acdS* (F: CGAAAGTGCTTTACGCGGAC and R: AGCCGTTGCGAAACAAGAAG) and PCR conditions as follows: 95°C (2min) – [95°C (20s) – 60°C (40s) – 72°C (1min) for 35cycles] – 72°C (5min). The amplified products were analyzed by electrophoresis on 1% (w/v) agarose gels.

### Production of T_2_ Generation Lines

The PCR-positive T_0_ lines (1, 7, and 20) and WT plants were grown in a greenhouse and self-pollinated to obtain T_1_ seeds. The obtained T_1_ seeds were screened *via in vitro* germination on a hormone-free MS basal medium containing 3.0% sucrose, 0.7% plant agar, and 1.0mg/L PPT. The culture conditions were the same as those described previously herein. Following this, the PPT-resistant T_1_ seeds were further grown in a greenhouse and self-pollinated to obtain the T_2_ homozygous lines. However, seeds of WT plants were germinated in plastic pots containing peat-based soil and self-pollinated to obtain their offspring.

### Cold Stress Treatment

Seeds of homozygous T_2_ lines (1, 7, and 20) and WT were germinated on hormone-free MS basal medium containing 3.0% sucrose and 0.7% plant agar. The culture conditions were the same as those described previously herein. After 4weeks of culture, the healthy seedlings of uniform sizes were sub-cultured on the same media under the same culture conditions for the next 4weeks. Before cold treatment, plant growth parameters of the 8-week-old plants (shoot length, number of leaves, leaf length, root length, and leaf width) were initially recorded, and the plants were discarded. Ten plants per line were used for this measurement, and this was repeated three times. Following this, the other plants were exposed to cold treatment in a growth chamber set at 8°C, with a 16h photoperiod and relative humidity of 60%, for 7days. There were 30 plants per line, with three replications. Leaf samples were collected from three independent plants per line during the cold stress period (0, 1, 3, 5, and 7days) for gene expression and other analyses, and the plants were discarded. Plant growth parameters were evaluated from 10 plants of each line at the end of the cold stress period, and this was repeated three times.

### Determination of Plant Growth After Recovery From Cold Stress

As described, during and after the cold stress treatment, some of the plants subjected to cold stress were used for the analysis of gene expression, plant growth parameters, and ethylene production. The remaining cold stress-treated plants were returned and grown under normal conditions [25°C±2°C and 16h photoperiod (intensity, 35μmolm^−2^ s ^−1^)]. Two weeks after growth under normal conditions, the recovery of the plants was investigated by determining the same plant growth parameters described previously herein. The determination contained 10 plants with three replications (a total of 30 plants).

### Drought Stress Treatment

*In vitro* 8-week-old homozygous transgenic (1, 7, and 20) and WT plants were transferred to plastic pots containing peat-based soil, and the soil was prepared with the same water content. After transplant to the pots for 1week under well-watered conditions, the plants were placed under drought conditions without watering for 1week. At the end of the treatment, the growth and physiological responses of the plants to drought stress were evaluated by determining stomatal density, chlorophyll content, relative water content (RWC), and fresh weight. In addition, leaf samples were collected from three independent plants per line during the drought stress period (0 and 7days) for analysis of ethylene production and its related gene expression, and 120 plants were used for each transgenic line and the WT plants.

### Measurement of Fresh Weight

The plants were removed from the pots, and the roots were thoroughly cleaned to be free of soil attached to the roots. Following this, the plants were immediately weighed using a portable microbalance. Ten different plants were used for each line, with three replicates each.

### Measurement of RWC

Post-drought-stress RWC was determined following the method described by Naing et al. (2017). The fifth leaves from the tops of the plants subjected to drought stress for 1week were used for this measurement. Leaves were collected from three different plants (three replicates per line), and the measurements were repeated three times.

### Determination of Stomatal Density

Leaf stomatal density was analyzed using the fifth leaves from the tops of the plants that were water deprived for 1week, as performed by Naing et al. (2017). Stomatal density was counted following the method of Chung et al. (2014). Leaves were collected from three different plants (three replications) per line, and the analysis was repeated three times.

### Determination of Chlorophyll Content

Following the stress, chlorophyll content was measured from the fifth leaves from the tops of the plants (T-1, T-7, T-20, and WT) using a chlorophyll meter (SPAD-502, Minolta). Leaves were collected from three different plants (three replications) per line, and the analysis was repeated three times.

### Determination of Plant Growth After Recovery

One week after water deprivation, some of the plants were used for the aforementioned experiments. Then, the remaining plants were regularly re-watered, and plant growth parameters (such as plant height, number of leaves, leaf length, and leaf width) were evaluated after 1week of re-watering. Fifteen plants per line were used for the analysis of plant growth, and there were three replicates for each line.

### Salt Stress Treatment

Similar to drought stress, *in vitro* 8-week-old homozygous transgenic (1, 7, and 20) and WT plants were transferred to plastic pots containing peat-based soil. The soil was prepared well to have the same water content. After transplanting them to the pots for 1week, the plants were watered every 3days with increasing sodium chloride (NaCl) salt concentrations (20, 30, 50, and 100mm). Twenty plants were used for each line, with three replicates each. Plants were maintained in a growth chamber set to 22°C and~60% relative humidity with a 16h photoperiod. Growth variables (plant height and fresh weight) were evaluated at the end of irrigation, where 10 plants per line with three replicates were used. At the end of the stress treatment (12days after treatment), three different leaves were sampled from each line for gene expression analysis.

### Expression Analysis of Cold-Regulated Gene, Antioxidant Genes, Proline Synthetic Gene, and Ethylene Biosynthetic Gene

The expression pattern of a cold-regulated gene (*CBF1*), antioxidant genes (*SOD* and *CAT*), proline synthetic gene (*Osmotin*), and ethylene biosynthetic gene (*ACO1*) in the leaves of plants exposed to cold stress for 0, 1, 3, 5, and 7days was, respectively, determined. The expression levels of *ACO1* in the leaves of plants exposed to drought stress for 0 and 7days and salt stress for 0 and 12days were determined, and those of *SOD*, *CAT*, and *Osmotin* in the leaves of plants exposed to salt stress for 12days were also determined. Total RNA was extracted from the leaves sampled, and reverse transcription was performed as described by Naing et al. (2017). The mRNA levels of the investigated genes were measured relative to those of the alpha-tubulin gene (reference gene) using the Real-Time PCR system (Thermo Fisher Scientific, Waltham, MA). Relative gene expression was calculated using the quantitative comparative CT method. The primers and PCR conditions used for the investigated genes are listed in Supplementary Table 1. Independent samples (three replicates) were used for the analysis of each line.

### Determination of Ethylene Production

Ethylene production in the fifth leaves from the tops of the plants subjected to cold and drought stresses for 0 and 7days was measured as described by Xu et al. (2020, 2021). For salt stress treatment, the production in the fifth leaf from the top of the plant was measured before (day 0) and after subjecting them to a final salt concentration (200mm, 12days). Briefly, approximately 100mg of leaves was sampled and immediately placed in a 50ml glass tube and sealed with a rubber septum. After 24h, ethylene production in the head space was measured using gas chromatography (GC-2010; Shimadzu, Tokyo, Japan). Three independent leaves (three replicates) per line were used for the analysis.

### Statistical Analysis

Data were statistically analyzed using SPSS version 11.09 (IBM Corporation, Armonk, United States) and are presented as means (of three replicates)±standard errors. Duncan’s multiple range test and the least significant difference test were used to separate the means, and the level of significance was set at *p*<0.05.

## Results

### Production of Transgenic *Petunia* Expressing the *acdS* Gene

**Figure 1:**
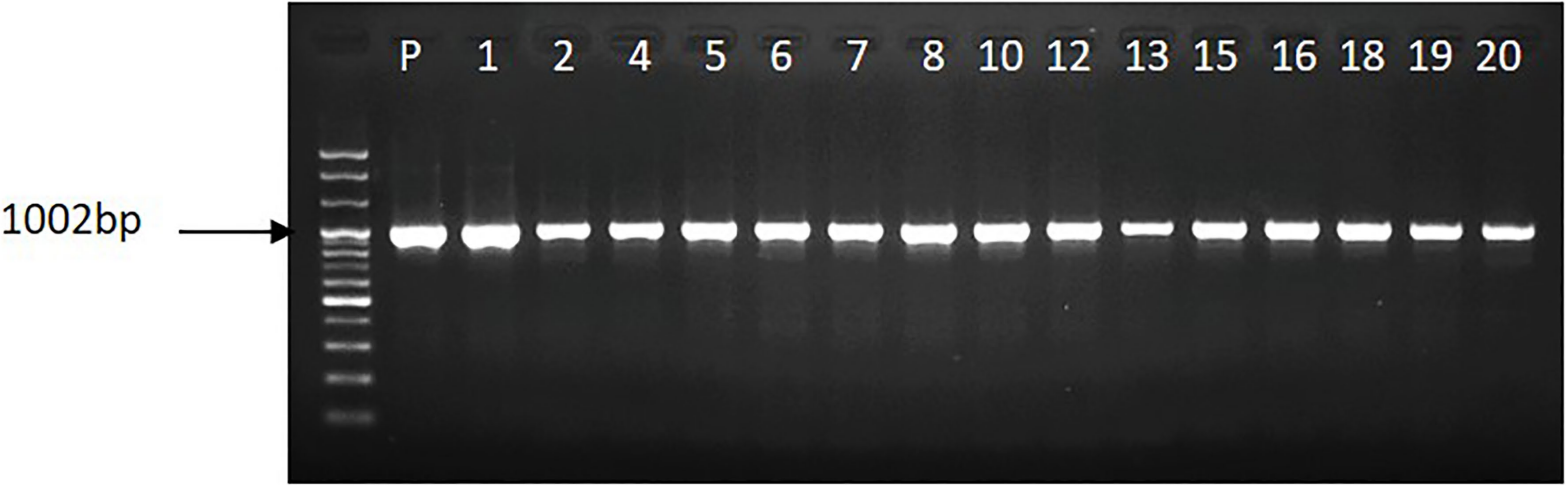
Illustration of the presence of *acdS* gene in the transgenic petunia cv. ‘Mirage Rose’, whereas p stands for plasmid.

The leaf explants infected with *Agrobacterium* suspension solution regenerated shoots on the selection media containing 0.5mgL^−1^ of PPT after 4weeks of culture. In total 20 PPT-resistant shoots were obtained from the 500 leaf explants, and most of the shoots (17 shoots) were able to produce roots and continuously survive well when cultured on hormone-free media containing 1.0mgL^−1^ PPT. Rooted plants were found to survive well when transferred to greenhouse conditions. PCR analysis revealed that the *acdS* gene was inserted in all rooted plants. The size of the amplified product (approximately 1,000bp) detected in the transgenic lines was identical to that observed in the plasmid PCB 302-3 used as the positive control (Figure 1, lane P). Among the transgenic lines obtained, the T_0_ lines (3, 7, and 20) were randomly selected and allowed to grow along with WT plants in the greenhouse until flowering. Homozygous progenies were obtained *via* successive self-pollination of the plants, and they were investigated for abiotic stress tolerance.

### Cold Stress Treatment

**Figure 2:**
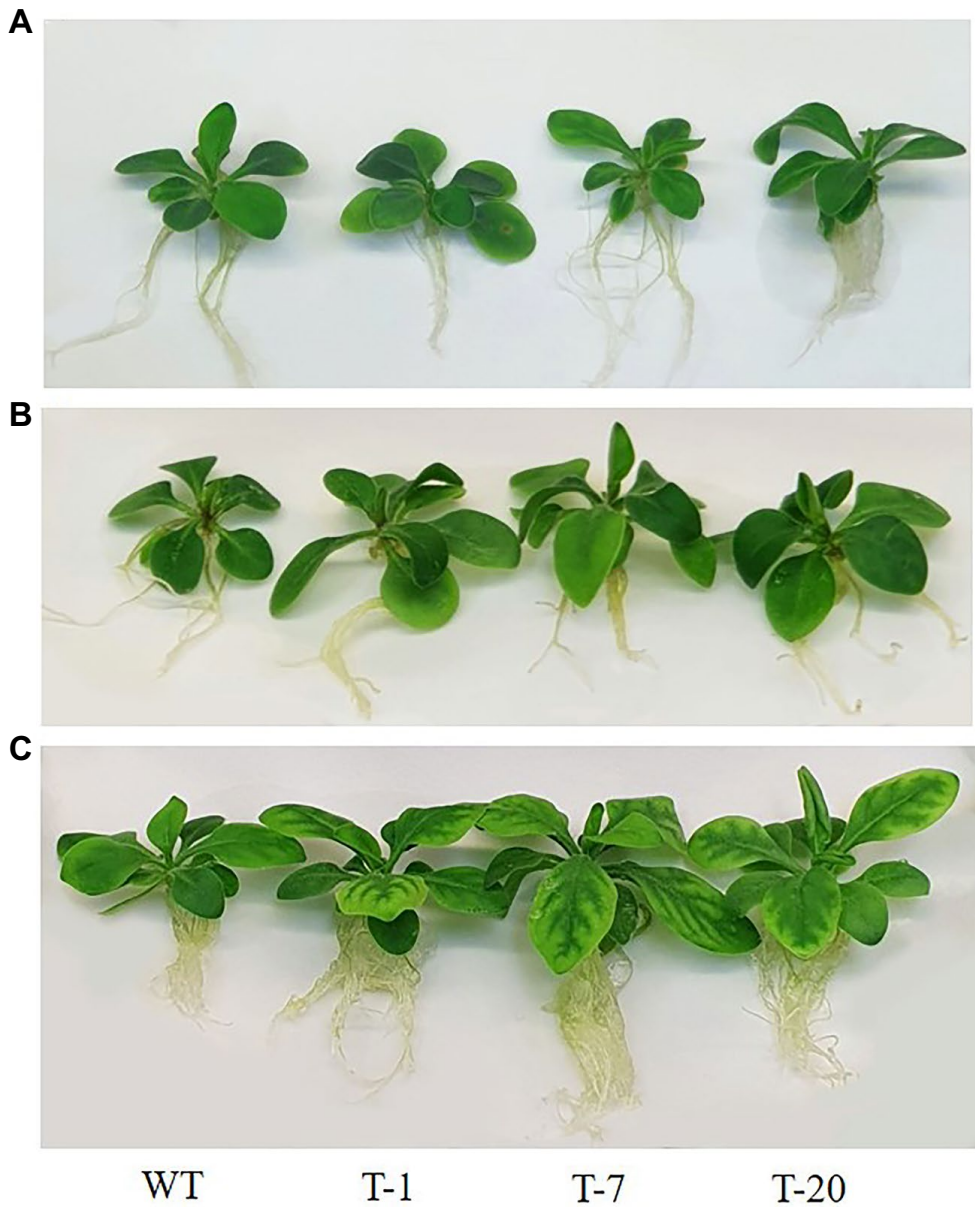
Illustration of growth status of wild type (WT) and transgenic petunia cv. ‘Mirage Rose’. **(A)** prior to cold stress, **(B)** end of cold stress, and **(C)** recovery from cold stress. From left to right (WT, T-1, T-7, and T-20).

**Figure 3:**
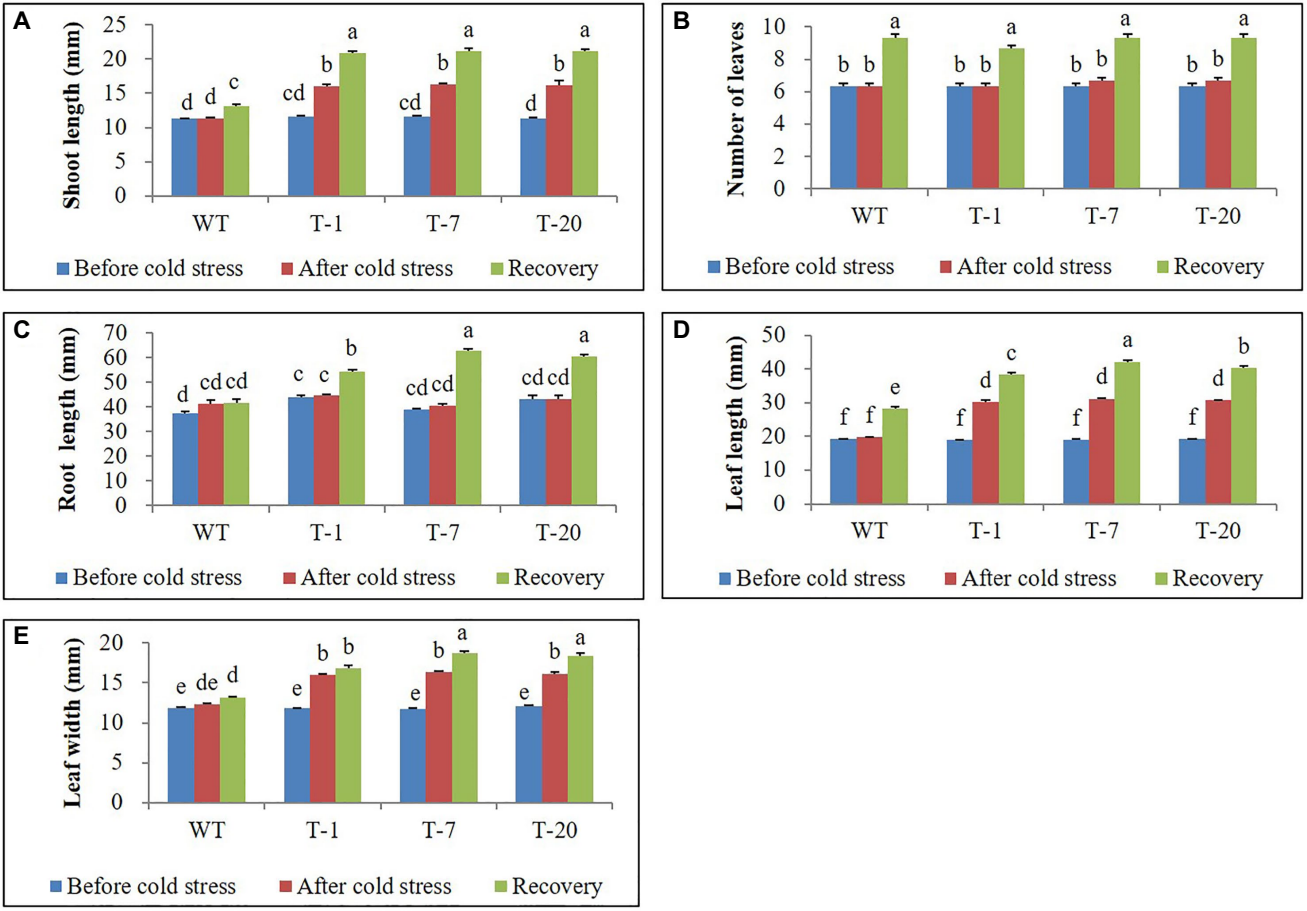
Illustration of growth parameters **(A–E)** of wild type (WT) and transgenic petunia cv. ‘Mirage Rose’, before cold stress, after cold stress, and recovery from cold stress, respectively. Data represent the means of three replicates, and error bars indicate standard error. Means with the same letters are not significantly different by Duncan’s Multiple Range Test (DMRT, *p*<0.05).

The transgenic and WT plants that did not show significant differences in plant growth were selected for cold stress treatment (Figures 2A, 3). When the plants were subjected to the cold stress condition for 7days, they suffered from stress and grew slower than those grown under normal conditions (at 22°C data not shown). Generally, WT plant growth was more influenced by cold stress as compared to that in the transgenic lines expressing *acdS* (Figure 2B), indicating more severe stress susceptibility in the former than in the latter. This was confirmed by determining the plant growth parameters (i.e., shoot length, leaf size, root length, and number of leaves), with significantly slower growth observed in the WT plants than in the transgenic plants (except for the number of leaves and root length; Figure 3). However, these differences were not significantly different among the transgenic plants. When the cold-treated plants were moved back to the normal condition for 7days, recovery from stress was clearly observed in all plants, particularly in the transgenic lines (Figures 2C, 3). Despite recovery in WT plants, the leaves were still smaller and the root length and shoot height were also relatively lower than those in the transgenic plants; thus, the plant growth parameters in the transgenic lines were significantly superior to those of WT (Figure 3). It was obvious that the overexpression of *acdS* in *Petunia* helped the plants tolerate cold stress to some extent and quickly recover from stress by promoting plant growth parameters (shoot length, leaf size, and number of leaves).

### Expression Analysis of Cold Regulatory Gene

**Figure 4:**
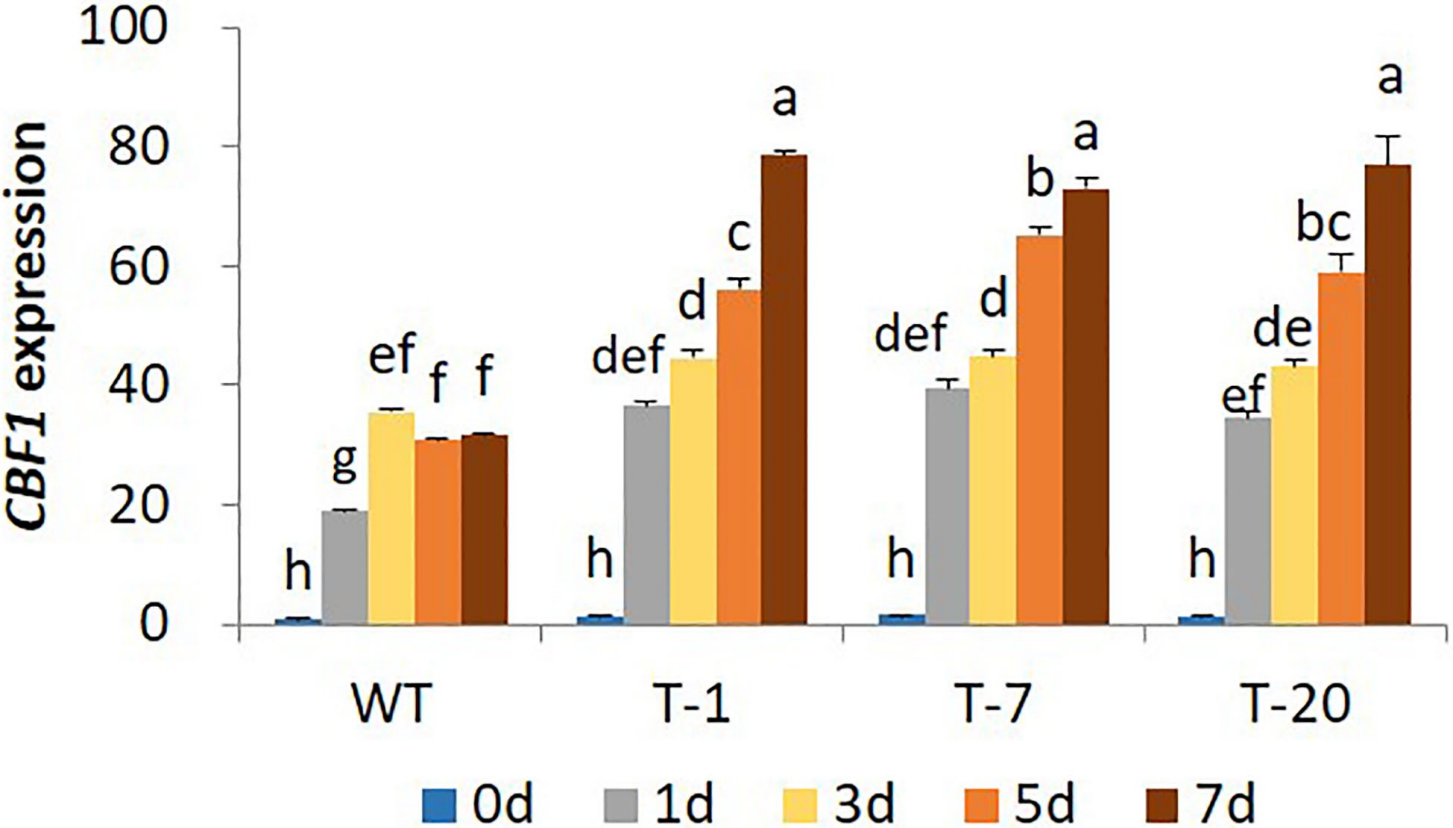
Illustration of transcript levels of cold-regulated gene *CBF1* gene expressed in wild type (WT) and transgenic petunia cv. ‘Mirage Rose’ during cold stress. Data represent the means of three replicates, and error bars indicate standard error. Means with the same letters are not significantly different by DMRT (*p*<0.05).

The higher cold stress tolerance in transgenic lines expressing *acdS* relative to that of WT was further confirmed by characterizing the expression levels of a cold regulatory gene (*CBF1*). Differential expression of *CBF1* in response to cold stress was clearly observed in WT and all transgenic plants because its expression levels were significantly higher in cold-treated plants (days 1, 3, 5, and 7) than in non-treated plants (day 0). In WT plants, an increase in its expression started on day 1 and reached a peak on day 3; however, it declined on day 5. However, its expression levels continuously increased from day 1 to 7 in the transgenic plants (Figure 4) despite a slight variation in the expression levels among the plants. In addition, the expression level of *CBF1* induced in WT plants during the cold stress period was lower than that in the transgenic plants.

### Drought Stress Treatment

**Figure 5:**
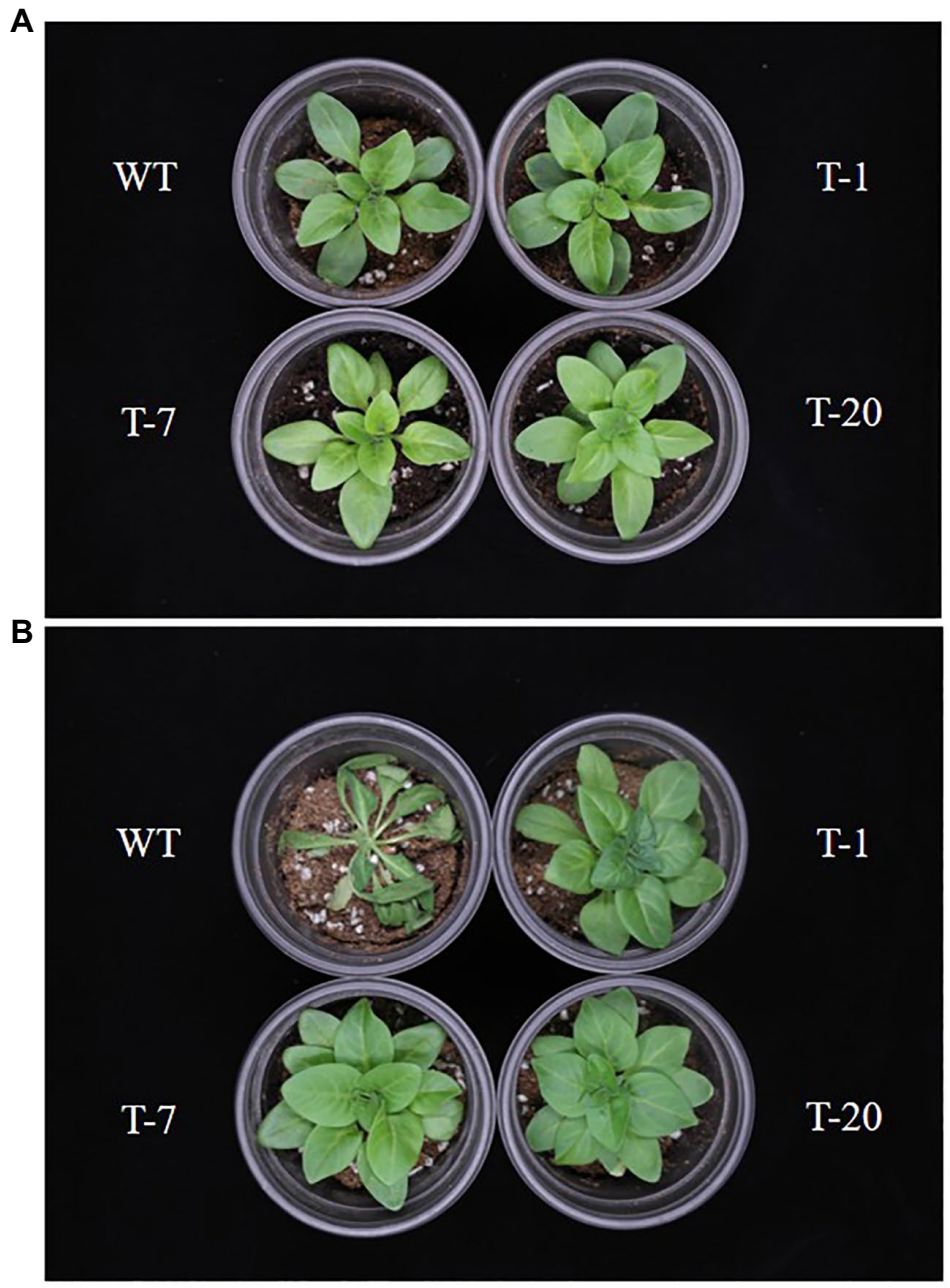
Illustration of growth status of wild type (WT) and transgenic petunia cv. ‘Mirage Rose’. **(A)** prior to drought stress and **(B)** end of the drought stress.

**Figure 6:**
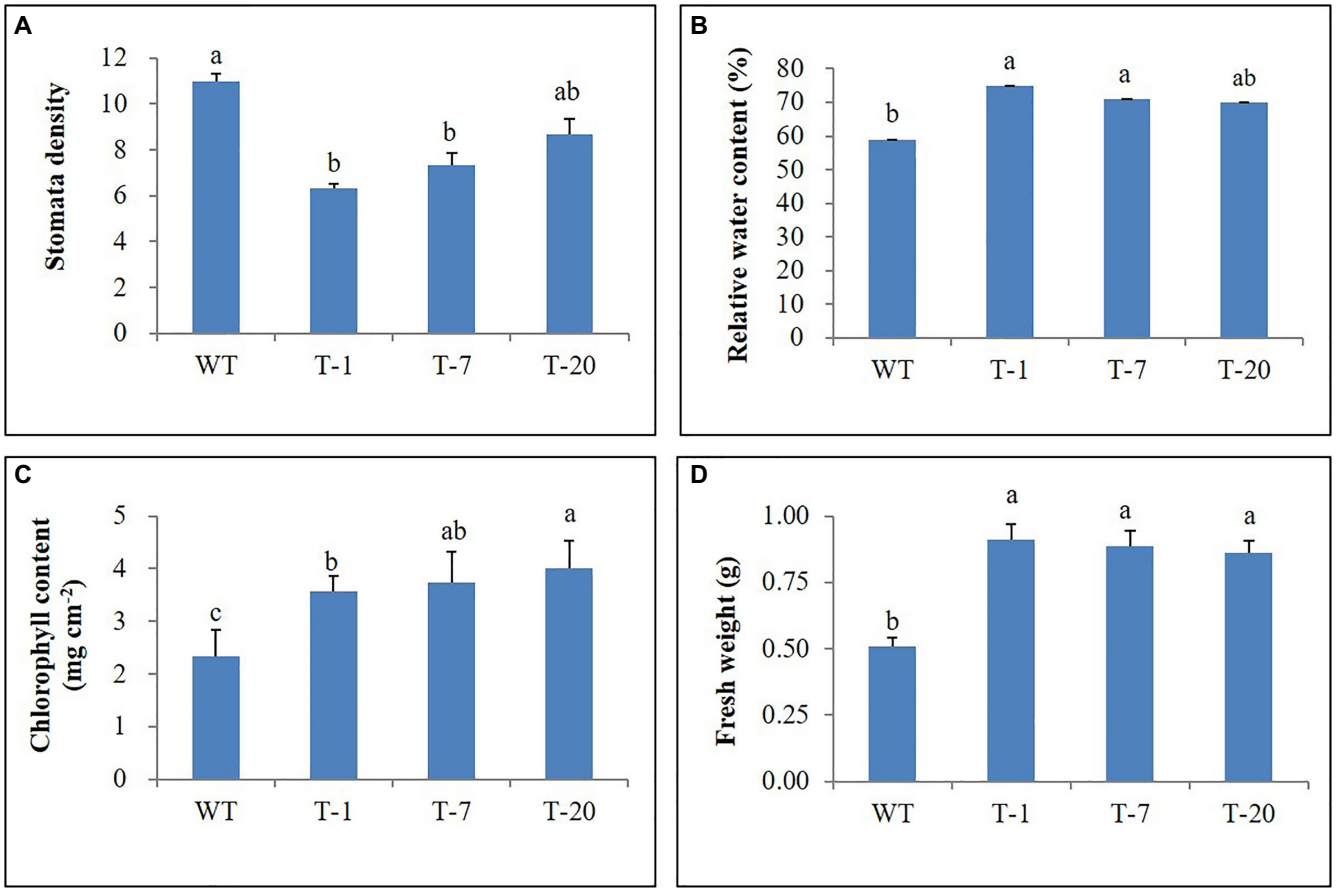
Comparison of physiological parameters **(A–D)** of wild type (WT) and transgenic petunia cv. ‘Mirage Rose’ following the drought stress. Data represent the means of three replicates, and error bars indicate standard error. Means with the same letters are not significantly different by Lest Significant Different Test (LSDT, *p*<0.05).

**Figure 7:**
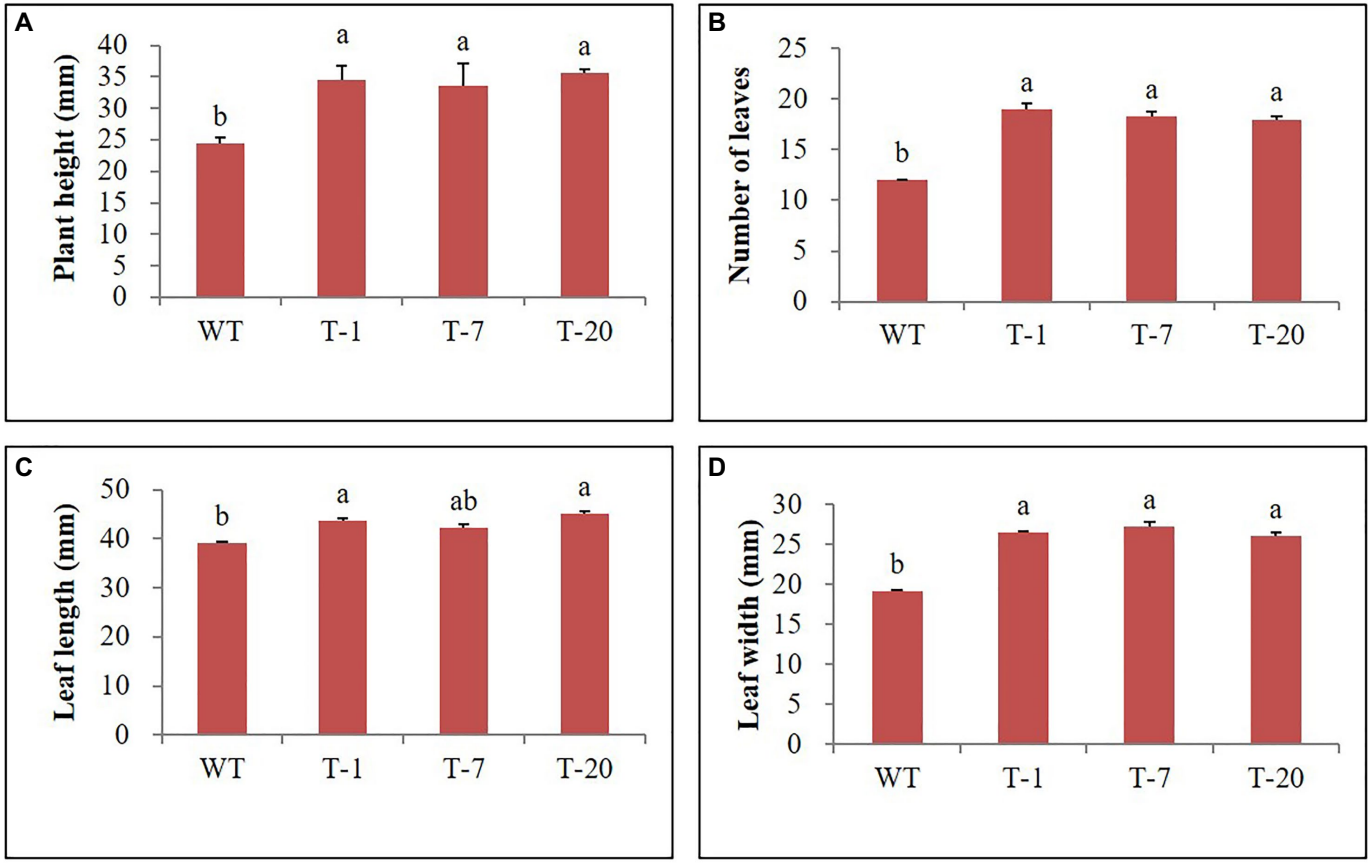
Illustration of growth parameters **(A–D)** of wild type (WT) and transgenic petunia cv. ‘Mirage Rose’ after recovery from drought stress. Data represent the means of three replicates, and error bars indicate standard error. Means with the same letters are not significantly different by LSDT (*p*<0.05).

Prior to being subjected to drought stress, the growth of transgenic and WT plants did not significantly differ (Figure 5A); however, following drought stress, almost all WT plant leaves appeared wilted. However, the leaves were not wilted, remained green, and fully expanded in transgenic plants (Figure 5B), which suggested tolerance to drought stress. To validate this, physiological parameters (i.e., leaf stomatal density, RWC, and chlorophyll content) and plant growth (i.e., fresh weight) linked to stress tolerance were assessed at the end of drought stress. When assessing leaf stomatal density, the number of stomata observed in WT plants was higher than that in transgenic plants (Figure 6A), which might lead to higher water loss in WT plants due to their high transpiration rates. As expected, RWC was found to be higher in transgenic plants than in WT plants (Figure 6B). Moreover, the results shown in Figure 6C also indicated the presence of higher chlorophyll content in transgenic plants than in WT plants. These results suggest that transgenic plants have higher drought stress tolerance that to WT plants. Higher fresh weight was also obtained from the transgenic plants (Figure 6D), confirming their sustained growth in response to drought stress. One week after re-watering, all WT and transgenic plants recovered from the stress. Recovery was faster and growth was better in transgenic plants than in WT plants, which was supported by the results of the plant growth parameters, such as plant height, leaf size, and number of leaves (transgenic plants>WT plants; Figures 7A–D).

### Salt Stress Treatment

**Figure 8:**
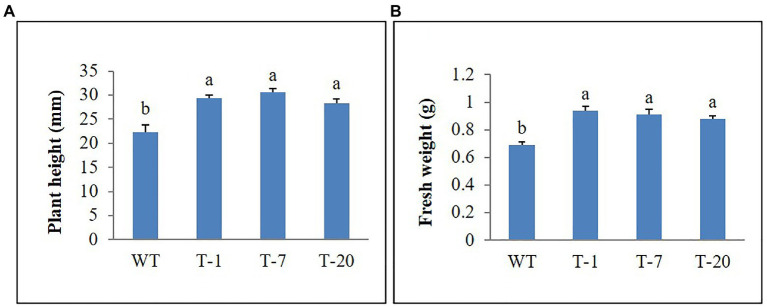
Comparison of growth parameters **(A)** and **(B)** of wild type (WT) and transgenic petunia cv. ‘Mirage Rose’ following salt stress. Data represent the means of three replicates, and error bars indicate standard error. Means with the same letters are not significantly different by LSDT (*p*<0.05).

The growth of transgenic and WT plants was not obvious following irrigation with water containing 20–50mm NaCl. However, upon watering with increasing NaCl concentrations (100mm), leaves started to curl and exhibited a decrease in shoot length. Under these conditions, WT plants were found to be more susceptible to stress than transgenic plants. This was proven by determining the plant height and fresh weight, with better results in the transgenic plants than in the WT plants (Figures 8A,B).

### Expression Levels of the Ethylene Biosynthetic Gene *ACO1* and Ethylene Production Under Cold, Drought, and Salt Stresses

**Figure 9:**
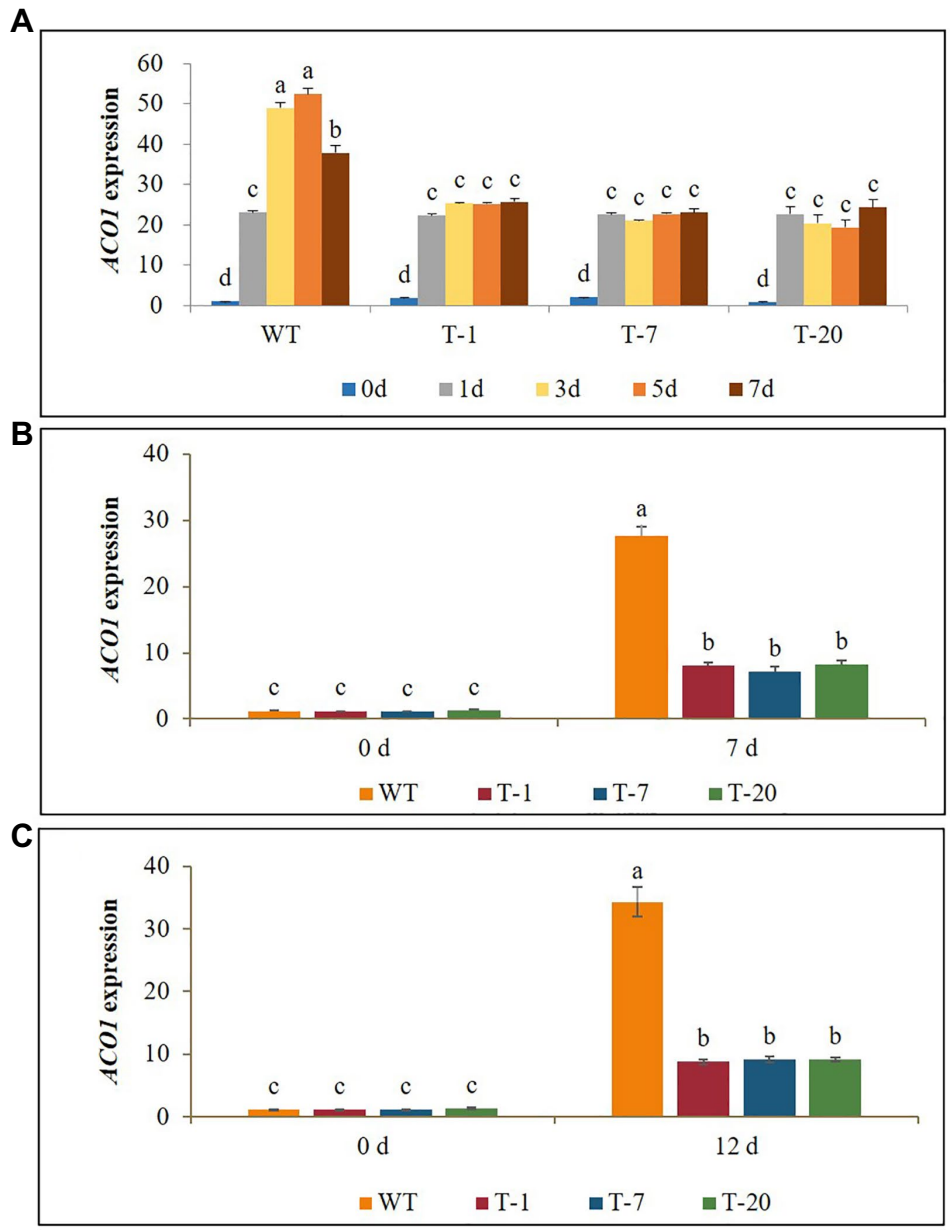
Illustration of transcript levels of ethylene biosynthesis gene *ACO1* expressed in wild type (WT) and transgenic petunia cv. ‘Mirage Rose’ exposed to cold stress for 0, 1, 3, 5, and 7days **(A)**, drought stress for 0 and 7days **(B)**, and salt stress **(C)** for 0 and 12days. Data represent the means of three replicates, and error bars indicate standard error. Means with the same letters are not significantly different by DMRT (*p*<0.05).

**Figure 10:**
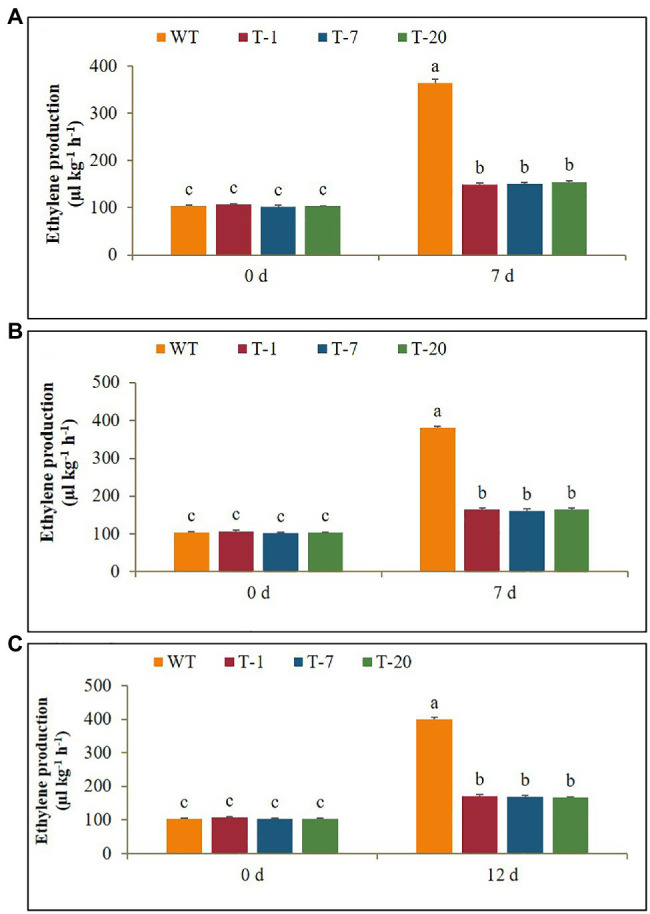
Comparison of ethylene production in wild type (WT) and transgenic petunia cv. ‘Mirage Rose’ exposed to cold stress **(A)** and drought stress **(B)** for 0 and 7days, and salt stress **(C)** for 0 and 12days. Data represent the means of three replicates, and error bars indicate standard error. Means with the same letters are not significantly different by DMRT (*p*<0.05).

Before subjecting plants to the stresses (cold, drought, and salt), *ACO1* expression levels between the WT and transgenic plants were not significantly different. When the WT plants were exposed to cold stress for 7days, a significant increase in *ACO1* expression was observed on day 1 after cold treatment. Their expression levels continuously increased and reached a peak on day 5, but a significant decrease was observed on day 7. An increase in *ACO1* expression caused by cold treatment was also observed in the transgenic lines. However, no further increase was observed even if the stress period was extended to day 7 (Figure 9A). This result was associated with higher production of ethylene in WT plants than in transgenic plants under stress conditions (Figure 10A). Overall, the lower *ACO1* expression and ethylene production were linked to higher cold stress tolerance in the transgenic plants. Similarly, the drought and salt stress tolerances were also linked to *ACO1* expression and ethylene production because the expression of *ACO1* and ethylene production were significantly higher in the WT leaves than in the transgenic leaves under drought stress (Figures 9B,C, 10B,C). It was obvious that the overexpression of *acdS* reduced stress-induced *ACO1* expression and ethylene production in the transgenic plants and could help the plants quickly recover from the stresses. This supports the hypothesis that plant tolerance to abiotic stress is associated with low ethylene production.

### Expression Analysis of Antioxidant-Related and Proline Synthetic Genes Under Cold and Salt Stresses

**Figure 11:**
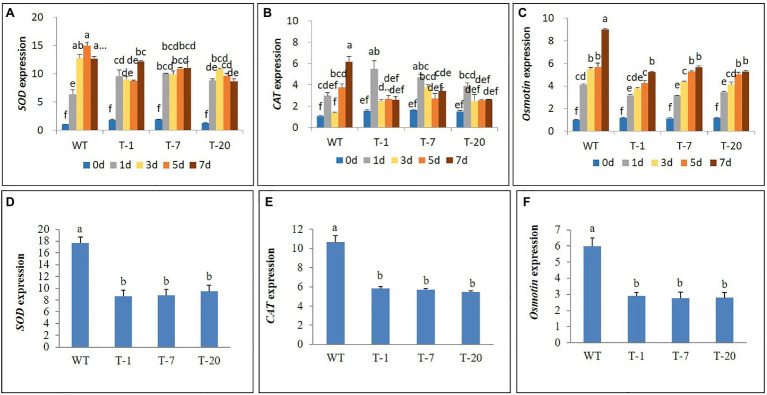
Illustration of transcript levels of antioxidant genes (*SOD* and *CAT*) and proline synthetic gene (*Osmotin*) expressed in wild type (WT) and transgenic petunia cv. ‘Mirage Rose’ exposed to cold stress for 0, 1, 3, 5, and 7days **(A–C)** and salt stress for 12days **(D–F)**. Data represent the means of three replicates, and error bars indicate standard error. Means (for cold stress) with the same letters are not significantly different by DMRT (*p*<0.05), and means (for salt stress) with the same letters are not significantly different LSDT (*p*<0.05).

The higher cold stress tolerance of transgenic lines expressing *acdS* relative to that of WT plants was further confirmed by characterizing the expression levels of antioxidant-related genes (*SOD* and *CAT*) and a proline synthetic gene (*Osmotin*). Elevated *SOD* expression levels caused by cold stress were observed in both WT and all transgenic plants in comparison with those in non-treated plants (day 0). The expression levels continued to increase from day 1 to 5 in WT plants but declined at day 7. In all transgenic plants, high induction of *SOD* by day 1 was observed. However, the expression levels were not significantly induced when they were exposed to cold stress for 3, 5, and 7days, except for in transgenic plants (T-1), which increased again on day 7. In general, WT plants induced higher expression levels of *SOD* during the cold stress period than transgenic plants; however, the expression levels were not significantly different except on day 5 (Figure 11A). High induction of *CAT* by day 1 was also observed in all plants, and particularly in the transgenic plants, but expression levels declined thereafter. In WT plants, significantly elevated expression occurred on day 7 (Figure 11B), and this was significantly higher than that in transgenic plants on day 7.

In addition, involvement of the *Osmotin* gene in the cold stress tolerance mechanism was also observed in all plants, because its expression levels in cold-treated plants were relatively higher than those in non-treated plants (day 0). Expression levels were positively associated with the length of stress periods in the WT plants. Although such an association was also observed in the transgenic lines, the expression levels in response to the cold stress period varied slightly depending on the transgenic plants (Figure 11C). Overall, cold stress-induced *Osmotin* gene expression levels were significantly higher in WT than in the transgenic lines, especially when the plants were subjected to cold stress for 7days. Similarly, when expression levels of *SOD*, *CAT*, and *Osmotin* in the leaves of plants exposed to salt stress for 12days were detected, the expression levels were also lower in transgenic plants than in WT plants under salt stress conditions (Figures 11D–F).

## Discussion

Owing to the continued deterioration of the global climate, it is expected that abiotic stresses will frequently occur and their durations would also be longer in the future; this might adversely affect plant growth and lead to a reduction in crop yield. The inhibition of plant growth mediated by stress is generally due to stress-induced ethylene and ROS overproduction (Bournonville and Diaz-Ricci, 2011; You and Chan, 2015; Assaha et al., 2017). ACC deaminase, which is encoded by the *acdS* gene, converts the ethylene biosynthetic enzyme (ACC) into ammonia and α-ketobutyrate and reduces ethylene production in higher plants (Honma and Shimomura, 1978). This was confirmed by the overexpression of *acdS* in some plant species, with a higher reduction in ethylene production and better plant growth in transgenic plants than in WT plants under abiotic stress (Reed et al., 1995; Sergeeva et al., 2006; Farwell et al., 2007; Zhang et al., 2008). However, the role of *acdS* in ethylene reduction and abiotic stress tolerance has not been investigated in *Petunia*, despite its importance as a bedding plant in the landscape industry and a model plant for biotechnology research. Hence, we overexpressed the *acdS* gene isolated from *P. veronii* in *P. hybrida* ‘Mirage Rose’. The role of *acdS* in ethylene reduction and plant growth under abiotic stresses was investigated using ethylene production, plant growth, physiological parameters, as well as expression levels of genes involved in ethylene production and stress tolerance.

Among the T_0_ plants (1-20) expressing the *acdS* gene, plants (1, 7, and 20) were randomly selected for the investigation of stress tolerance mechanisms. Before starting the investigation, T_2_ generations of the selected T_0_ lines (1, 7, and 20) and WT plants were obtained *via* successive self-pollination and screening. In our study, when the transgenic and WT plants were subjected to the abiotic stresses (cold, drought, and salt), the plants suffered and grew slower than those grown under normal conditions. This might be because ethylene production in the plants under the stress conditions could be higher than that under normal conditions. This is because the cold stress-induced ethylene precursor ACC (Concellon et al., 2005; Hershkovitz et al., 2009) and the negative effects of ACC induction on plant growth have been reported (Shi et al., 2012; Ali et al., 2014). In addition, drought stress-induced ethylene production and its negative effects on plant growth in various plant species have also been demonstrated (Graves and Gladon, 1985; Sharp, 2002; Balota et al., 2004). Moreover, salt stress also induces ethylene in many plant species (Bar et al., 1998; Botella et al., 2000; Alvarez et al., 2003; Arbona et al., 2005; Nadeem et al., 2010; Maxton et al., 2018; Tiwari et al., 2018), and the induced ethylene adversely affects plant growth (Alvarez et al., 2003; Maxton et al., 2018; Tiwari et al., 2018). However, the growth of WT plants was more retarded than that of transgenic plants under the various stresses. The greater stress tolerance of transgenic plants over WT plants was proven as they exhibited better plant growth performance and physiological parameters. Under drought stress, the greater tolerance of transgenic plants over WT plants was associated with an enhanced reduction in stomatal numbers, higher RWC, and higher chlorophyll content in transgenic plants. The greater reduction in the stomatal number will control the high transpiration rate and maintain the RWC to sustain the growth of transgenic plants under stress. A reduction in chlorophyll content is also one of the major responses to early drought stress, and this impairs the photosynthesis system of plants and negatively affects plant growth. In this study, the higher chlorophyll content in transgenic plants than in WT plants also strongly supported the greater tolerance of transgenic plants to stress, wherein a higher chlorophyll content would markedly help photosynthesis to sustain growth in response to stress. Wang et al. (2012) observed that cucumber plants inoculated with PGPB exhibit a higher chlorophyll content and stress tolerance than non-inoculated plants. Naing et al. (2017) observed that drought-tolerant plants contain a higher RCW and lower stomata density than drought-susceptible plants under drought stress. Recently, Maxton et al. (2018) and Saikia et al. (2018) reported that pepper and pulse plants inoculated with *acdS*-containing PGPB maintain a higher chlorophyll content than non-inoculated plants and exhibit tolerance against salt and drought stresses.

The better growth, superior physiological parameters, and greater tolerance of transgenic plants relative to WT plants could be mainly due to the lower production of stress-induced ethylene in the former than in the latter. As expected, ethylene production and expression of *ACO1* in transgenic plants were significantly lower than those observed in WT plants under stress conditions. The resulting lower ethylene production and *ACO1* expression in the former might be due to the overexpression of *acdS* because it encodes ACC deaminase, the activities of which can easily break down the stress-induced ACC to form α-ketobutyrate and ammonia (Glick et al., 1998; Glick, 2014), thus reducing the expression of *ACO1* and ethylene production. PGPB that express the *acdS* gene have been employed to alleviate the deleterious effects of drought- and salt-induced ethylene in various plant species (Barnawal et al., 2014; Jaemsaeng et al., 2018; Maxton et al., 2018; Saleem et al., 2018; Tiwari et al., 2018; Gupta and Pandey, 2020; Zhang et al., 2020), whereas lower ethylene production, higher plant height, and higher biomass were observed in the inoculated plants. Sergeeva et al. (2006) and Farwell et al. (2007) also claimed that transgenic canola overexpressing *acdS* could tolerate flooding and high salt stress. Moreover, transgenic expression of *acdS* in *Arabidopsis* alleviates the adverse effects of salt and water-logging stress (Jung et al., 2018). In this study, a greater reduction in the stomatal number and ethylene production, a higher RWC, and a higher chlorophyll content in transgenic plants made them less susceptible to ethylene stress to sustain their growth under stress. Therefore, compared to that with WT plants, the recovery of transgenic plants from drought was quicker, indicating that ethylene had a less deleterious effect on the transgenic plants.

Under cold stress, cold-induced *ACO1* expression was significantly higher in WT than in transgenic plants during the stress period. The continued increase of *ACO* expression in WT to 5days would cause the plant to suffer from high ethylene production, leading to greater growth retardation. In contrast, despite the cold-induced expression of these genes in transgenic plants, its expression level was not relatively high and did not increase even when the stress period was extended to 7days. According to the gene expression results, the *acdS* gene effectively reduced *ACO1* expression until day 7. Consequently, this would result in low ethylene production and would improve the growth of the plants under stress. A similar role for the *acdS* gene in improving cold stress tolerance and plant growth has been demonstrated in grapevine (Barka et al., 2006; Theocharis et al., 2012). Subramanian et al. (2015) also reported that tomato plants inoculated with *acdS*-containing bacteria have reduced expression of *ACO* and ACC and ACO activities under cold stress.

In addition, compared to those in transgenic plants, expression levels of the cold-regulated gene *CBF1* were also lower in WT plants. It is possible that WT plants suffered more from the cold-induced ethylene than transgenic plants owing to the higher expression of *ACO1* in WT plants than in transgenic plants; consequently, higher levels of *CBF1* were not triggered to protect against cold stress. A reduction in *CBF1* expression mediated by cold stress-induced ethylene production has been reported previously (Zhao et al., 2014). Shi et al. (2012) also observed that ethylene negatively regulates freezing tolerance by suppressing *CBF* expression in *Arabidopsis*. In addition, increased expression of *CBF* in tomato inoculated with *acdS*-expressing bacteria has also been reported (Subramanian et al., 2015). Therefore, this result suggests that the retardation of plant growth under cold stress is positively associated with the effect of cold stress-induced ethylene and the suppression of *CBF1* by cold stress-induced ethylene. Since WT plants suffered from cold-induced ethylene stress more severely than transgenic plants, the recovery of WT plants was slower than that of transgenic plants following transfer to normal growing conditions.

Abiotic stress not only induces ethylene but also ROS, which are toxic to plants (Jaemsaeng et al., 2018; Gupta and Pandey, 2020). In this study, it seemed that cold and salt stresses also induced ROS because differential expression of *SOD*, *CAT*, and *Osmotin* was observed in the plants during stress. Generally, the expression was lower in transgenic plants than in WT plants. It seemed that the WT plants suffered more from stress than the transgenic plants, because the transgenic plants could be less damaged by ethylene-induced ROS owing to the overexpression of *acdS*. This might allow the transgenic plants to express genes that reduce and scavenge stress-induced ROS to a higher level than that in WT plants for sustained growth. Maxton et al. (2018) and Saikia et al. (2018) observed higher antioxidant and proline activities in pepper and pulse plants inoculated with *acdS*-containing PGPB relative to those in non-inoculated plants under abiotic stress. The involvement of antioxidant and proline activities in the abiotic stress (cold and salt)-induced ROS scavenging and stress tolerance mechanism has been well demonstrated (Mittler et al., 2004; Zhang et al., 2006; Xu et al., 2010; Mohammadian et al., 2012; Jaemsaeng et al., 2018; Naing et al., 2018; Kyu et al., 2019; Gupta and Pandey, 2020; Naing and Kim, 2021).

## Conclusion and Future Prospect

This study has demonstrated that the overexpression of *acdS* in *Petunia* improves tolerance to various abiotic stresses, such as cold, drought, and salt, compared to that in WT plants. These tolerances were proven by showing better plant growth and physiological performance in the transgenic plants than in WT plants. Moreover, expression analysis of the genes involved in the stress tolerance mechanism also supported the improved stress tolerance in transgenic plants. These stress tolerance mechanisms were linked to ethylene production in the plants. Moreover, the overexpression of *acdS* significantly reduced ethylene production in the transgenic plants and improved plant growth in response to abiotic stresses as compared to those with WT plants. These results suggest that the overexpression of *acdS* reduces stress-induced ethylene production and improves plant growth under abiotic stress. Therefore, the findings described in this study can contribute to the genetic improvement of other ornamental plants that can tolerate various abiotic stresses. In the future, abiotic stresses will frequently occur and their durations could also be longer owing to continued deterioration of the global climate. The use of floricultural plants overexpressing *acdS* might increase in the landscape industry as they can overcome the deleterious effects of abiotic stress-induced ethylene, which adversely affects plant growth. Therefore, the generation of transgenic plants expressing the *AcdS* gene might be more popular in the future to continue to support development of the floricultural industry.

## Data Availability Statement

The datasets presented in this study can be found in online repositories. The names of the repository/repositories and accession number(s) can be found in the article/Supplementary Material.

## Author Contributions

AN designed the experiments, wrote the manuscript, and revised the manuscript. AN and HJ conducted the experiments. SJ assisted the experiments. CK supervised the project. All authors contributed to the article and approved the submitted version.

## Funding

This work was carried out with the support of Cooperative Research Program for Agriculture Science & Technology Development (Project No. PJ01485801) Rural Development Administration, Republic of Korea.

## Conflict of Interest

The authors declare that the research was conducted in the absence of any commercial or financial relationships that could be construed as a potential conflict of interest.

## Publisher’s Note

All claims expressed in this article are solely those of the authors and do not necessarily represent those of their affiliated organizations, or those of the publisher, the editors and the reviewers. Any product that may be evaluated in this article, or claim that may be made by its manufacturer, is not guaranteed or endorsed by the publisher.
