# Supplementary material for: Overexpression of 1-Aminocyclopropane-1-Carboxylic Acid Deaminase (acdS) Gene in Petunia hybrida Improves Tolerance to Abiotic Stresses
Source: Front Plant Sci. 2021 Nov 2;12:737490. doi: 10.3389/fpls.2021.737490 (PMC8594826; doi:10.3389/fpls.2021.737490)
Supplement: Supplementary file 1 [file Table_1.DOCX]

Supplementary Table 1. Primers used for gene expression analysis using quantitative real-time PCR.

| Gene  (Accession No.) | | Primers sequences | |
| --- | --- | --- | --- |
|  |  | Forward primer **(5’ to 3’)** | Reverse primer **(5’ to 3’)** |
| *ACO1* | L21976.2 | 5'- ATCAGCTTGGACAAAGTGAATGG-3' | 5'-CACCAACTCAAAGAAGCCCC-3' |
| *CBF1* | (GO_dr004P0029M08_F_ab1) | ATATGGCTGAAGGGCTAA  -3' | ACTAGAAGAATTAGACAGAATAACC |
| *SOD* | X14352.1 | ACTCAGTCGTTGGAAGAGCG  -3' | TGGTAAGGCTGAGTTCGTGG |
| *CAT* | AY726007.1 | CAGCCAGTGGGACGATTAGT | GGCACCACAATAGAAGGGCA |
| *Osmotin* | AF376058.1 | CTTTCGCCCCAACTAAGCCT | TGCACCAGGACATTCACCAT |
| *Tublin* | SGN-U207876 | 5'-TGGAAACTCAACCTCCATCCA-3' | 5'-TTTCGTCCATTCCTTCACCTG-3' |

*TUB: tubulin gene (Mallona et al., 2010).

*ACO1*, 1-aminocyclopropane-1-carboxylate oxidase 1; *SOD*; superoxide dismutase, *CAT*, catalase; *CBF1*; C-repeat binding factor 1; *Osmotin*; Osmotin; Tublin, b-Tubulin 6 chain used as an internal standard.

PCR condition - 95℃(10min) followed by 40 cycles of [95℃(15s)-53.9℃(1min)] -95℃(15s)-60℃(1min)-95℃(15s)
